# The Genomic Epidemiology of Clinical *Burkholderia pseudomallei* Isolates in North Queensland, Australia

**DOI:** 10.3390/pathogens13070584

**Published:** 2024-07-15

**Authors:** Ian Gassiep, Mark D. Chatfield, Budi Permana, Delaney Burnard, Michelle J. Bauer, Thom Cuddihy, Brian M. Forde, Johanna Mayer-Coverdale, Robert E. Norton, Patrick N. A. Harris

**Affiliations:** 1 UQ Centre for Clinical Research, Faculty of Medicine, The University of Queensland, Royal Brisbane and Women’s Hospital Campus, Herston, Brisbane, QLD 4029, Australia; m.chatfield@uq.edu.au (M.D.C.); b.permana@imb.uq.edu.au (B.P.); michelle.j.b@hotmail.com (M.J.B.); b.forde@uq.edu.au (B.M.F.); johannak.mayer@gmail.com (J.M.-C.); p.harris@uq.edu.au (P.N.A.H.); 2 Department of Infectious Diseases, Mater Hospital Brisbane, Brisbane, QLD 4101, Australia; 3 Pathology Queensland, Royal Brisbane and Women’s Hospital, Herston, Brisbane, QLD 4029, Australia; 4 Queensland Cyber Infrastructure Foundation, Brisbane, QLD 4067, Australia; delaney.burnard@qcif.edu.au; 5 Institute for Molecular Bioscience (IMB), The University of Queensland, Brisbane, QLD 4067, Australia; t.cuddihy1@uq.edu.au; 6 Sullivan Nicolaides Pathology, Brisbane, QLD 4006, Australia; 7 Herston Infectious Diseases Institute, Royal Brisbane and Woman’s Hospital, Herston, Brisbane, QLD 4029, Australia; 8 Pathology Queensland, Townsville University Hospital, Townsville, QLD 4814, Australia; robert.norton@health.qld.gov.au; 9 Faculty of Medicine, The University of Queensland, Brisbane, QLD 4006, Australia

**Keywords:** *Burkholderia pseudomallei*, melioidosis, whole genome sequencing, multilocus sequence type, virulence factors

## Abstract

**Background:** *Burkholderia pseudomallei*, the causative agent of melioidosis, is highly genetically recombinant, resulting in significant genomic diversity. Multiple virulence factors have been associated with specific disease presentations. To date, there are limited data relating to genomic diversity and virulence factors associated with melioidosis cases in North Queensland, Australia. **Aim:** To describe the genetic diversity of *B. pseudomallei* and identify virulence factors associated with clinical risk factors and patient outcomes. **Methods:** Whole genome sequencing of clinical isolates was performed and analysed with clinical data obtained from a retrospective melioidosis cohort study. **Results:** Fifty-nine distinct sequence types (STs) were identified from the 128 clinical isolates. Six STs comprised 64/128 (50%) isolates. Novel STs accounted for 38/59 (64%) STs, with ST TSV-13 as the most prevalent (n = 7), and were less likely to possess an LPS A genotype or YLF gene cluster (*p* < 0.001). These isolates were most likely to be found outside the inner city (aOR: 4.0, 95% CI: 1.7–9.0, *p* = 0.001). ST TSV-13 was associated with increased mortality (aOR: 6.1, 95% CI: 1.2–30.9, *p* = 0.03). Patients with a history of alcohol excess were less likely to be infected by *fhaB*3 (aOR 0.2, 95% CI: 0.1–0.7, *p* = 0.01) or YLF (aOR: 0.4, 95% CI: 0.2–0.9, *p* = 0.04) positive isolates. **Conclusions:** There are a significant number of novel sequence types in Townsville, Australia. An emerging novel ST appears to have an association with geographic location and mortality. Ongoing investigation is required to further understand the impact of this ST on the Townsville region.

## 1. Introduction

*Burkholderia pseudomallei* is a saprophytic environmental organism found predominantly in the soil and water of tropical and subtropical regions. The organism is endemic in multiple regions of Australia, including Western Australia, The Northern Territory, and Queensland [1].

*B. pseudomallei* is the causative pathogen of melioidosis, an opportunistic infectious disease that most commonly affects patients with risk factors such as diabetes mellitus, immunosuppression, or excess alcohol intake [1]. Acquisition of infection is most commonly through direct inoculation or inhalation, and the most common manifestations of disease are pneumonia and bacteraemia [1]. In Australia, the case-fatality rate ranges from 6 to 23% [2,3,4].

Although the *B. pseudomallei* genome is highly recombinant, whole genome sequencing (WGS) has provided significant insights into both its epidemiology and the genetic association of disease manifestations [5,6]. Multilocus sequence typing (MLST) is a genotyping method used to characterise the genetic diversity of *B. pseudomallei*. This method identifies seven housekeeping genes, provides each allele with a number, and uses this string of numbers to create a sequence type (ST) [7]. This is a rapid, portable, and robust method of comparing genomic populations of *B. pseudomallei*, both within a specific region and globally. It has been used to attribute an outbreak’s point-source and also to analyse geographic distribution [8,9], as it has been noted that STs are often clustered within a 50 km radius [9].

Whole genome sequencing has become more accessible, and as such, global genomic data regarding both clinical and environmental *B. pseudomallei* isolates has increased [10,11,12,13]. Regions with limited prior sequencing data have reported high proportions of novel sequence types. Two studies from India reported that 65% and 94% of isolates were identified as novel STs, respectively [10,13]. A Malaysian study reported that 40% of STs identified were novel [12].

Genomic data have also improved our understanding of virulence factors and disease manifestation. The *Burkholderia* intracellular motility factor A variant (*bimA*_Bm_) was found to be associated with neurological disease, and the filamentous hemagglutinin gene (*fhaB*3) associated with bacteraemia [14,15]. Yersinia-like fimbriae (YLF) and *Burkholderia thailandensis*-like flagellum and chemotaxis (BTFC) gene clusters are reported to have distinct geographic distribution, and the YLF gene cluster is reportedly associated with clinical, as opposed to environmental, isolates [16].

A recent study analysed the genomic epidemiology of *B. pseudomallei* isolates across a large proportion of Queensland, Australia [17]. A significant number of these cases were located in the city of Townsville, for which the authors have detailed clinical data [4]. Therefore, the authors aimed to use the combination of these data to describe the local genomic diversity and analyse the association between clinical data and genomic risk factors for melioidosis in this region.

## 2. Ethical Approval

This study received ethical approval from the Royal Brisbane and Women’s Hospital Ethics Committee (LNR/2020/QRBW/65573), with site-specific authority obtained from the Townsville Hospital and Health Service and approval under the Queensland Public Health Act.

## 3. Methods

Townsville University Hospital (TUH) is a 742-bed tertiary referral centre in North Queensland serving a local population of approximately 195,000 inhabitants. All patients ≥ 18 years of age with culture-confirmed melioidosis identified between 1 January 1997 and 31 December 2020 were included. Retrospective clinical details were obtained from hospital medical records.

One bacterial isolate from each patient was prospectively collected and stored during the study period. Isolates were recovered from −80 °C storage, subcultured onto horse blood agar (HBA), and assessed for purity. DNA extraction was performed with the QIAGEN DNAeasy ultra-pure DNA extraction kit according to the manufacturer’s instructions. Sequencing libraries were generated using the Nextera Flex DNA library preparation kit and sequenced on the NovaSeq6000 (Illumina Inc., San Diego, CA, USA) on an S1 flow cell, generating 150-bp paired-end reads according to the manufacturer’s instructions. Data generated from this study are available under the NCBI accession PRJNA960936.

Raw Illumina reads were trimmed with Trimmomatic v0.36 [18]. Read quality was assessed with multiQC v1.11 and genomes were assembled with SPAdes v3.14.0 [19,20]. Reads were mapped with BWA-MEM v0.7.17 [21]. Sequence types (STs) were determined with multilocus sequence typing (MLST) (https://github.com/tseemann/mlst) and reads were mapped to alleles retrieved from pubMLST (access date: 25 April 2023) [22]. Whole genome alignment (4,950,632 bp) and phylogenomic analysis were performed using parSNP v1.7.4 and IQtree v2.1.2 (1000 bootstrap replicates), respectively [23,24,25].

A custom database was created and operated with ABRicate v1.0.1 (https://github.com/tseemann/abricate) [26] to screen for virulence determinants, including *Burkholderia* intracellular motility factor A (*bim*A), lipopolysaccharide (LPS) profile, filamentous hemagglutinin proteins (*fhaB3*), Yersinia-like fimbriae (YLF), and *Burkholderia thailandensis*-like flagellum and chemotaxis (BTFC) gene clusters, and detection of the *bim*A allele variant [14,27]. Reference strains for these virulence factors are provided in Supplementary Table S1.

### Statistical Analysis

Data were analysed using Stata version 16 (StataCorp, College Station, TX, USA). Categorical variables were analysed using the chi-squared test or Fisher’s exact test. The Cochrane–Armitage test was used to analyse trends over time. A threshold of *p* ≤ 0.1 in a simple logistic regression model was used for inclusion of a covariate in the multivariate logistic regression model.

## 4. Results

### 4.1. Multilocus Sequence Type

**Figure 1:**
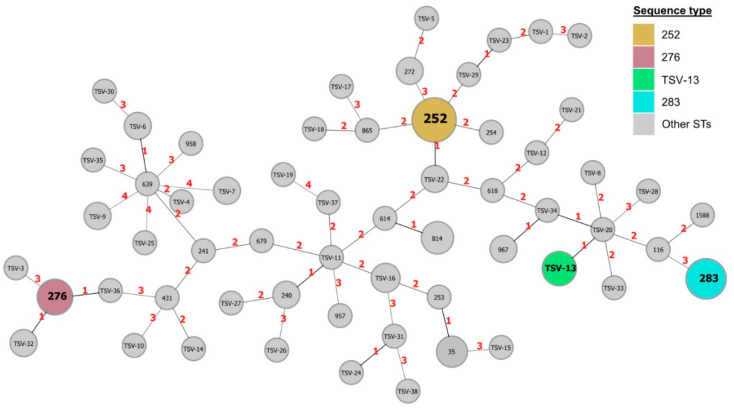
eBURST diagram of all clinical *B. pseudomallei* isolates. Each circle represents an individual sequence type (ST). Diameter sizes correspond to the number of isolates within each ST. Red numbers depict the number of locus variants between STs.

Fifty-nine distinct sequence types were identified from 128 clinical isolates, and six STs comprised 64/128 (50%) isolates. Of those identified with pubMLST, ST 252 was the most prevalent (26/128, 20%), followed by ST 283 (15/128, 12%), and ST 276 (8/128, 6%). Novel or not previously described STs, labelled as TSV (Townsville), accounted for 38/59 (64%) STs identified and comprised 51/128 (40%) of clinical isolates. ST TSV-13 was the most prevalent (7/38), followed by ST TSV-16 (3/38) (Figure 1).

**Figure 2:**
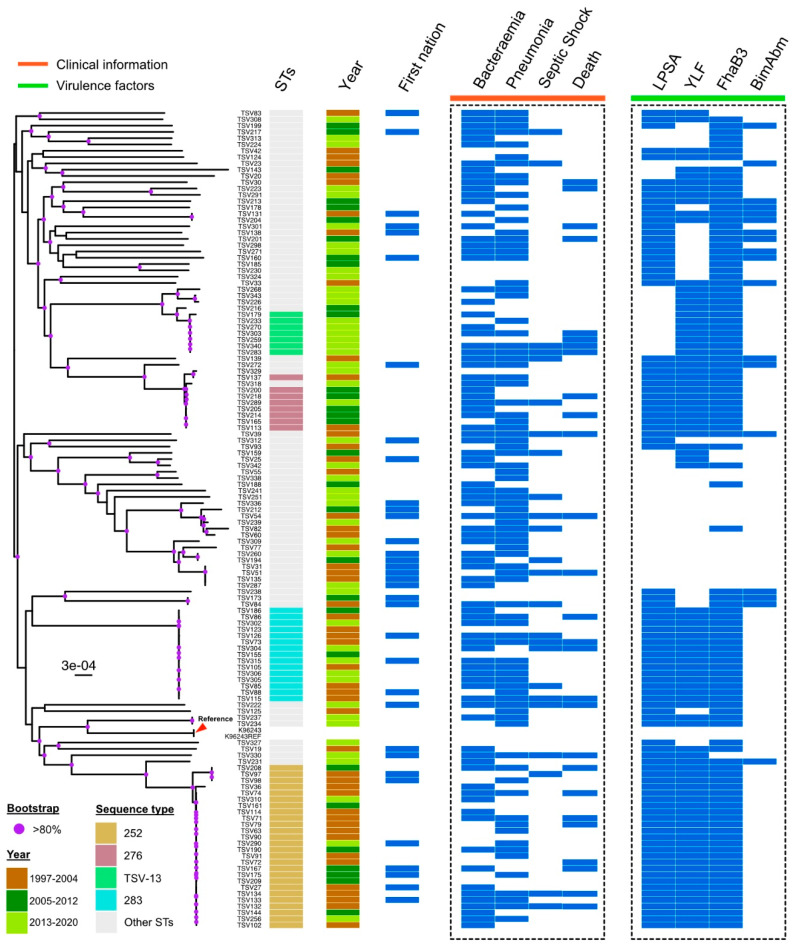
Phylogenetic tree of all clinical isolates with associated metadata.

**Figure 3:**
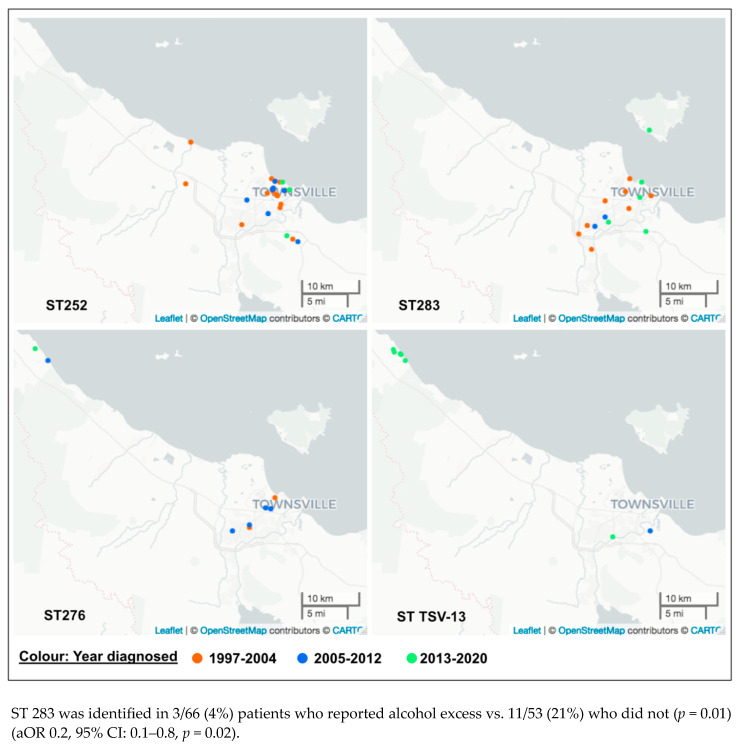
Geographic distributions of the four most common sequence types over time.

Over the course of the study period there was a significant change in sequence types identified (Table 1, Figure 2). The prevalence of novel STs increased from 10/50 (20%) of all isolates between 1997 and 2004 to 28/48 (58%) between 2013 and 2020 (*p* = 0.001), while the most prevalent previously identified sequence type, ST 252, decreased from 16/50 (32%) to 3/48 (6%), and the most prevalent novel sequence type, ST TSV-13, increased from 0/50 to 6/48 (13%) (*p* < 0.001) (Figure 3 and Supplementary Table S2). Furthermore, the geographical locations of these sequence types appear to have changed over time (Figure 3).

An earlier study examined the geospatial distribution of culture-positive melioidosis in a similar area of Townsville. This covered the period 1996 to 2008. Sixty-five cases were identified, and 38% of these covered the area around the base of a central hill, Castle Hill [28]. The study reported here, with cases occurring after this period, did not confirm the predominance of sites of acquisition as being in suburbs around Castle Hill.

The majority of cases, 111/128 (87%), occurred during the wet season (November–April). No clinical nor genomic association was identified upon multivariate analysis.

When assessing sequence types in relation to clinical data, there was an association between ST and mortality. The four most common STs had a higher case-fatality rate compared to all other STs and an adjusted odds ratio (aOR) of 3.0 (95% CI: 1.2–7.9, *p* = 0.02) (Supplementary Table S3), as did ST TSV-13 compared to all other STs (aOR: 6.1, 95% CI: 1.2–31, *p* = 0.03). There was no difference in STs in patients who identified as First Nations (FNs) Australians.

### 4.2. Virulence Factors

Of the specific virulence factors assessed, *fhaB*3 was present in 105/128 (82%) isolates, YLF in 88/128 (69%), BTFC in 40/128 (31%), and *bimA*_Bm_ in 18/128 (14%). In terms of lipopolysaccharide in silico serotyping, LPS A was most prevalent, in 89/128 (70%), and 39/128 (30%) could not be classified. No serotype B or B2 was identified.

A total of 87 patients were bacteraemic. There were no virulence factors associated with bacteraemia, including *fhaB*3, which was present in 70/87 (80%) bacteraemic patients and 32/37 (86%) non-bacteraemic patients (*p* = 0.42) (Supplementary Table S4). Pneumonia was present in 84 patients, with no associated virulence factor. Additionally, no association was identified between any other clinical presentation and the genomic factors assessed (Supplementary Tables S4–S7). In terms of risk factor analysis, patients with a history of excess alcohol intake were less likely to be infected with *fhaB*3 positive (aOR 0.2, 95% CI: 0.1–0.7, *p* = 0.01) or YLF positive isolates (aOR: 0.4, 95% CI: 0.2–0.9, *p* = 0.04) (Supplementary Tables S4 and S5).

Geographic population analysis demonstrated no association between a suburb location and age, sex, or First Nations status. With regard to genomic factors, there were associations with location. Novel sequence type isolates were most likely to be found outside inner city suburbs, even when adjusting for age and FNs status (aOR: 4.0, 95% CI: 1.7–9.0, *p* = 0.001). In contrast, the LPS A genotype was least likely to occur in the outer city region (−18°22” and −19°38S to 145°93 and 146°93”E) (aOR 0.2, 95% CI: 0.1–0.8, *p* = 0.01). The novel sequence type TSV-13 was also associated with this outer city region (*p* = 0.007) (Figure 3). This particular region of interest includes a higher proportion of elderly residents. The average number of residents between 60 and 79 years of age is twice the state and national average. All patients infected with this ST were over 50 years of age. Three of seven (43%) were 50–69, and 4/7 (57%) were older than 70 years (*p* = 0.05).

In terms of virulence factors, TSV STs were less likely to possess an LPS A genotype (28/51 (55%) vs. 61/77 (79%), *p*= 0.003) or the YLF gene cluster (26/51 (51%) vs. 62/77 (81%), respectively, *p* < 0.001) (Table 1). Note that upon multivariate analysis, only the LPS A genotype remained statistically significant. No ST TSV-13 isolate harboured the LPS A genotype. This ST represented 7/39 (18%) of non-LPS A isolates (*p* < 0.001) (Supplementary Table S2).

The case-fatality rate for those patients infected with an isolate containing the YLF gene cluster was 27% compared to 10% for those infected with the BTFC gene cluster (aOR 3.2, 95% CI: 0.9–10.8, *p* = 0.06). The LPS A genotype, *fhaB3*, and *bimA*_Bm_ were not associated with increased mortality (Supplementary Table S3).

## 5. Discussion

This study provided an analysis of whole genome sequencing data from a comprehensive dataset of *B. pseudomallei* clinical isolates from the Townsville region of North Queensland. *B. pseudomallei* is genetically diverse; however, strains of the organism are geospatially clustered [29]. As expected, we described a substantial number of novel *B. pseudomallei* sequence types, with 38 STs not previously reported.

Prior studies have demonstrated a correlation between relative abundance and the composition of environmental and clinical STs [6]. In The Northern Territory, Australia, more than 450 STs have been described. However, in the Darwin region, only five STs comprise 90% of the sequence type abundance [29]. In contrast, our data demonstrate six STs in the Townsville region comprising only 50% of clinical isolates.

It is interesting to note the change in STs over time, with a significant increase in novel types. This is likely partially explained by the previous lack of sequencing data from this region. The increased prevalence of ST TSV-13 may be an early sign of a dominant Townsville sequence type. This is potentially alarming given the possible association of this sequence type with mortality. Although the specific region most affected by this sequence type has a substantially older population, the adjusted odds ratio for mortality remained elevated. Additionally, the 10-year population growth rate for this area was 18% compared with 8% for Townsville overall. Similarly, the number of dwellings increased by 27% compared to 12%, respectively [30]. Finally, this ST was first identified in 2009, and not more than once per year. Taken together, these data suggest that ST TSV-13 may have a specific geographic environmental niche, and that it was not associated with a point-source outbreak of melioidosis. Given demographic changes over time, it is likely this ST will result in more infections.

The ST TSV-13 isolates and more recent cases of melioidosis are now found scattered in newer northern suburbs (unpublished data). Reasons for this changing epidemiology can only be speculative at this point. Different soil types, disturbance of soil due to new building excavations, and increasing populations of people with risk factors living in these areas are all potential explanations.

With regard to the virulence factors assessed in this study, our data contrast those previously reported for *fhaB*3. We did not find a positive association with this factor and bacteraemia, nor a negative association with cutaneous disease [14]. Unfortunately, the association between *bimA*_Bm_ and neurological disease could not be examined due to the low number of clinical cases. However, this has been well described [31].

The YLF/BTFC gene clusters were previously reported as having two geographical distributions [16]. The YLF gene cluster is predominant in Thailand (98%), while 79% of Australian isolates from The Northern Territory possess the BTFC cluster [14,16]. Additionally, no clinical correlation has been identified with these gene clusters. Data from our study add to international epidemiological data, as 68% of isolates from Townsville contained the YLF gene cluster, which differs from both The Northern Territory and Thai data. There was a possible association with mortality and the YLF gene cluster in our cohort, although this was not significant upon multivariate analysis.

Current *B. pseudomallei* lipopolysaccharide in silico data suggest that 87% of Australian isolates and 97% of Thai isolates are LPS genotype A [27,32]. Additionally, LPS B was identified in 12% and 2% of isolates, respectively [27,32]. LPS B2, the rarest genotype, has not been reported in Thai isolates, and was not detected in our study. Similar to our data, the Australian LPS genotypes were not associated with bacteraemia, septic shock, or mortality. However, in contrast to both regions, we report a lower prevalence of LPS A (70%) and a significant number of isolates that were not classified. Although there was no clear correlation between these isolates and clinical data, this may have been due to the sample size. Additional analysis of these isolates and their sequences is required to determine both LPS phenotype and genotype.

In terms of diagnosis, LPS B is known to generate a greater immune response compared to LPS A [33]. Data presented in this study may provide some insight into the previously reported low seropositivity amongst melioidosis patients in Townsville [34]. However, a greater number of clinical isolates with associated serological data are needed to further assess this relationship.

The reason for the association between variable virulence factor-negative strains and patients with alcohol excess remains unclear. This may be a sign of a susceptible host infected with a less virulent organism. Although there was no association between these virulence factors and other features of immunosuppression, this may have been due to the sample size. Additional research into host factors, such as cytokine response, and bacterial in vivo transcriptomic analysis, may provide insights into associations demonstrated in this study.

This study was limited to analysis of previously well-described variable virulence factors. As such, it is possible that virulence factors not assessed in this dataset may be associated with both clinical manifestations of disease and patient outcomes. Clinical data were obtained from a retrospective study; therefore, not all risk factors were verifiable. Finally, although there appears to be an association between ST type and mortality, this is a small dataset upon which many analyses were undertaken, and further genomic analysis of *B. pseudomallei* isolates from this region is required.

## 6. Conclusions

This study demonstrated a high level of *B. pseudomallei* genomic diversity within Townsville, North Queensland. A substantial number of novel STs were identified. An emerging novel ST appears to be associated with geographic location, and possibly with mortality. WGS data also revealed an unexpected variation in YLF/BTFC gene clusters, which have previously represented distinct geographical regions. These data add to local and international genomic epidemiology. However, further research into both environmental and future clinical *B. pseudomallei* isolate genomic data is required.

## Figures and Tables

**Table 1:** Novel sequence types and their relationship with clinical data and virulence factors.

			Novel ST N (%)	Bivariate *p*-Value	aOR (95% CI)	Multivariate *p*-Value
**Time period**	1997–2004		10/40 (20)	<0.001	Ref	
	2005–2012		13/30 (43)		3.8 (0.9–16.1)	0.07
	2013–2020		28/48 (58)		9.1 (2.5–32.9)	0.001
**Clinical data**						
Age > 50 y		Yes	33/91 (36)	0.2		
		No	18/37 (49)			
Sex		Male	36/88 (44)	0.3		
		Female	15/46 (33)			
First Nations		Yes	13/34 (38)	0.7		
		No	35/81 (43)			
Alcohol excess		Yes	30/66 (45)	0.5		
		No	20/53 (38)			
Diabetes mellitus		Yes	23/59 (39)	1.0		
		No	26/65 (40)			
Chronic kidney disease		Yes	5/7 (71)	0.1	3.1 (0.4–20.9)	0.2
		No	42/107 (39)			
Malignancy		Yes	5/16 (31)	0.6		
		No	44/106 (42)			
Lung disease		Yes	16/37 (43)	0.7		
		No	33/87 (38)			
No risk factors		Yes	2/13 (15)	0.1		
		No	47/112 (42)			
Bacteraemia		Yes	36/87 (41)	0.55		
		No	13/37 (35)			
Pneumonia		Yes	33/84 (39)	0.68		
		No	16/37 (43)			
Solid organ abscess		Yes	5/15 (33)	1.0		
		No	42/103 (41)			
Skin and soft tissue		Yes	7/17 (41)	1.0		
		No	42/103 (41)			
Genitourinary		Yes	8/19 (42)	1.0		
		No	41/102 (40)			
Septic shock		Yes	38/90 (42)	1.0		
		No	10/24 (42)			
**Virulence factors**						
LPS A		Yes	28/89 (31)	0.003	0.2 (0.1–0.6)	0.004
		No	23/39 (59)			
YLF		Yes	26/88 (30)	<0.001	0.8 (0.3–2.2)	0.6
		No	25/40 (62)			
*fhaB*3		Yes	41/105 (39)	0.69		
		No	10/23 (43)			
*bimA*_Bm_		Yes	16/18 (89)	<0.001	41.2 (6.7–251)	<0.001
		No	35/110 (32)			

## Data Availability

Data generated from this study are available under the NCBI accession PRJNA960936.

## Supplementary Materials

The following supporting information can be downloaded at: https://www.mdpi.com/article/10.3390/pathogens13070584/s1, Table S1: Reference strains used to create custom virulence factor database; Table S2: Clinical data and virulence factors of the most prevalent sequence types; Table S3: Association between genomic factors and mortality; Table S4: Bivariate and multivariate associations with *fhaB3*. Table S5: Bivariate and multivariate associations with YLF. Table S6: Bivariate and multivariate associations with LPS A. Table S7: Bivariate and multivariate associations with *bim*ABm.
