# Supplementary material for: The Genomic Epidemiology of Clinical Burkholderia pseudomallei Isolates in North Queensland, Australia
Source: Pathogens. 2024 Jul 15;13(7):584. doi: 10.3390/pathogens13070584 (PMC11279585; doi:10.3390/pathogens13070584)
Supplement: Supplementary file 1 [file pathogens-13-00584-s001.zip › pathogens-3025253-supplementary.pdf]

Supplementary Table 1. Reference strains used to create custom virulence factor database

**Virulence factors**	**Reference strain**	**Locus tag/accession**
YLF	*B. pseudomallei* K96243 chromosome 2	BPSS0120
YLF	*B. pseudomallei* K96243 chromosome 2	BPSS0123
YLF	*B. pseudomallei* K96243 chromosome 2	BPSS0122
YLF	*B. pseudomallei* K96243 chromosome 2	BPSS0121
LPS_B	*B. pseudomallei* 579	NZ_ACCE01000003
LPS_A	*B. pseudomallei* K96243 chromosome 1	NC_006350
LPS_B2	*B. pseudomallei* MSHR840	GU574442
$bimA_{Bm}$	*B. pseudomallei* 668	BURPS668_A2118
BTFC_cluster	*B. pseudomallei* 305	EF377328.1
fhaB3	*B. pseudomallei* K96243 chromosome 2	BPSS2053

Supplementary Table 2. Clinical data and virulence factors of the most prevalent sequence types

			Total, N	ST 252<br>N (%)	ST 283<br>N (%)	ST 276<br>N (%)	ST TSV-13<br>N (%)	Other STs<br>N (%)
**Time period**	1997-2004		50	16 (32)	8 (16)	2 (4)	0	24 (48)
	2005-2012		30	7 (23)	2 (7)	5 (17)	1 (3)	15 (50)
	2013-2020		48	3 (6)	5 (10)	1 (2)	6 (13)	33 (69)
**Clinical data**	Age >50y	Yes	91	20 (22)	12 (13)	7 (8)	7 (8)	45 (49)
		No	37	6 (16)	3 (8)	1 (3)	0	27 (73)
	Sex	Yes	82	14 (17)	9 (11)	6 (7)	6 (7)	47 (57)
		No	46	12 (26)	6 (13)	2 (4)	1 (2)	25 (54)
	First Nation	Yes	34	7 (21)	3 (9)	0	0	24 (71)
		No	81	16 (20)	11 (14)	4 (5)	7 (9)	43 (53)
	Alcohol excess	Yes	66	14 (21)	3 (5)	4 (6)	5 (8)	40 (61)
		No	53	9 (17)	11 (21)	2 (4)	2 (4)	29 (55)
	Diabetes mellitus	Yes	59	11 (19)	7 (12)	4 (7)	2 (3)	35 (59)
		No	65	14 (22)	8 (12)	3 (5)	5 (8)	35 (54)
	Chronic kidney disease	Yes	7	1 (14)	0	0	1 (14)	5 (71)
		No	107	21 (20)	15 (14)	6 (6)	6 (6)	59 (55)
	Malignancy	Yes	16	2 (13)	3 (19)	3 (19)	1 (6)	7 (44)
		No	106	23 (22)	12 (11)	3 (3)	6 (6)	62 (59)
	Lung disease	Yes	37	8 (22)	5 (14)	4 (11)	1 (3)	19 (51)
		No	87	18 (21)	10 (11)	3 (3)	6 (7)	50 (57)
	No risk factors	Yes	13	5 (39)	3 (23)	0	1 (8)	4 (31)
		No	112	21 (19)	12 (11)	8 (7)	5 (4)	66 (59)
	Bacteraemia	Yes	87	15 (17)	12 (14)	7 (8)	5 (6)	48 (55)
		No	37	10 (27)	3 (8)	1 (3)	2 (5)	21 (57)
	Pneumonia	Yes	84	14 (17)	11 (13)	5 (6)	4 (5)	50 (60)
		No	37	11 (30)	3 (8)	1 (3)	3 (8)	19 (51)
	Solid organ abscess	Yes	15	2 (13)	4 (27)	0	1 (7)	8 (53)
		No	104	22 (21)	10 (10)	6 (6)	5 (5)	61 (59)
	Skin & soft tissue	Yes	17	4 (24)	3 (18)	0	1 (6)	9 (53)
		No	103	20 (19)	11 (11)	6 (6)	5 (5)	60 (58)
	Genitourinary	Yes	19	4 (21)	3 (16)	1 (5)	2 (11)	9 (47)
		No	102	20 (20)	12 (12)	5 (5)	5 (5)	60 (59)
	Septic Shock	Yes	24	3 (13)	5 (21)	1 (4)	2 (8)	13 (54)
		No	90	18 (20)	9 (10)	5 (6)	5 (6)	53 (59)
**Virulence factors**	LPS A	Yes	89	26 (29)	15 (17)	8 (9)	0	40 (45)
		No	39	0	0	0	7 (18)	32 (82)
	YLF	Yes	88	26 (30)	15 (17)	8 (9)	7 (8)	32 (36)
		No	40	0	0	0	0	40 (100)
	*fhaB*3	Yes	105	26 (25)	15 (14)	8 (8)	7 (7)	49 (47)
		No	23	0	0	0	0	23 (100)
	$bimA_{Bm}$	Yes	18	0	0	0	0	18 (100)
		No	110	26 (24)	15 (14)	8 (7)	7 (6)	54 (49)

Supplementary Table 3. Association between genomic factors and mortality

		Dead<br>N (%)	Bivariate p-value	aOR (95% CI)	Multivariate p-value
**Novel ST**	Yes	9/51 (18)	0.5	0.7 (0.3 – 1.9)	0.5
	No	18/77 (23)			
**YLF/BTFC**	YLF	23/88 (26)	0.04	3.2 (0.9 – 10.8)	0.06
	BTFC	4/40 (10)			
***FhaB*3**	Yes	24/105 (23)	0.4	1.9 (0.5 – 7.8)	0.4
	No	3/23 (13)			
**LPS A**	Yes	21/89 (24)	0.4	1.4 (0.5 – 4.0)	0.5
	No	6/39 (15)			
***BimA*$_{Bm}$**	Yes	2/18 (11)	0.2	0.5 (0.1 – 2.0)	0.3
	No	25/110 (23)			
**Sequence Types**	ST252	8/26 (31)	0.02	3.0 (1.2 – 7.9)	0.02
	ST283	4/15 (27)			
	ST276	2/8 (25)			
	ST TSV13	4/7 (57)			
	Other STs*	9/72 (13)			

*All STs excluding 4 most common

Supplementary Table 4. Bivariate and multivariate associations with *fhaB*3

	*fhaB*3 +ve	Bivariate p-value	aOR (95% CI)	Multivariate p-value
	**N (%)**			
**Age >50**				
Yes	82/91 (90%)	<0.001	1.7 (0.3 – 8.9)	0.5
No	23/37 (62%)			
**Sex**				
Female	38/46 (83%)	0.9		
Male	67/82 (82%)			
**First Nation**				
Yes	20/34 (59%)	<0.001	0.3 (0.1 – 1.3)	0.1
No	73/81 (90%)			
**Alcohol excess**				
Yes	47/66 (71%)	0.001	0.2 (0.1 – 0.7)	0.01
No	50/53 (94%)			
**Diabetes mellitus**				
Yes	47/59 (80%)	0.6		
No	54/65 (83%)			
**Chronic Kidney disease**				
Yes	6/7 (86%)	1		
No	88/107 (82%)			
**Malignancy**				
Yes	15/16 (94%)	0.3		
No	84/106 (79%)			
**Lung disease**				
Yes	31/37 (84%)	0.8		
No	70/87 (80%)			
**No Risk Factors**				
Yes	13/13 (100%)	0.1		-
No	90/112 (80%)			
**Pneumonia**				
Yes	68/88 (77%)	0.05	0.4 (0.1 – 1.6)	0.2
No	34/37 (92%)			
**Bacteraemia**				
Yes	70/87 (80%)	0.4		
No	32/37 (86%)			
**Solid organ abscess**				
Yes	14/15 (93%)	0.3		
No	82/104 (79%)			
**Skin & Soft tissue**				
Yes	16/17 (94%)	0.2		
No	81/103 (78%)			
**Genitourinary**				
Yes	15/19 (79%)	0.8		
No	83/102 (81%)			
**Septic Shock**				
Yes	17/24 (71%)	0.2		
No	76/90 (84%)			

Supplementary Table 5. Bivariate and multivariate associations with YLF

	YLF	Bivariate p-value	aOR (95% CI)	Multivariate p-value
	**N (%)**			
**Age >50**				
Yes	70/91 (77%)	0.002	2.7 (0.7 – 9.9)	0.1
No	18/37 (49%)			
**Sex**				
Female	33/46 (72%)	0.6		
Male	55/82 (67%)			
**First Nation**				
Yes	17/34 (50%)	0.01	0.8 (0.2 – 3.2)	0.8
No	60/81 (74%)			
**Alcohol excess**				
Yes	39/66 (59%)	0.02	0.4 (0.2 – 0.9)	0.04
No	42/53 (79%)			
**Diabetes mellitus**				
Yes	40/59 (68%)	0.7		
No	46/65 (71%)			
**Chronic Kidney disease**				
Yes	5/7 (71%)	1		
No	73/107 (68%)			
**Malignancy**				
Yes	12/16 (75%)	0.8		
No	72/106 (68%)			
**Lung disease**				
Yes	28/37 (76%)	0.4		
No	58/87 (67%)			
**No Risk Factors**				
Yes	12/13 (92%)	0.1		-
No	75/112 (67%)			
**Pneumonia**				
Yes	58/88 (66%)	0.3		
No	28/37 (76%)			
**Bacteraemia**				
Yes	62/87 (71%)	0.7		
No	25/37 (68%)			
**Solid organ abscess**				
No	72/104 (69%)	0.6		
Yes	9/15 (60%)			
**Skin & Soft tissue**				
Yes	9/15 (60%)	0.3		
No	68/103 (66%)			
**Genitourinary**				
Yes	14/19 (74%)	0.8		
No	69/102 (68%)			
**Septic Shock**				
Yes	16/24 (67%)	1		
No	61/90 (68%)			

Supplementary Table 6. Bivariate and multivariate associations with LPS A

	LPS A +ve	Bivariate p-value	aOR (95% CI)	Multivariate p-value
	N (%)			
**Age >50**				
Yes	67/91 (74%)	0.1	1.8 (0.8 – 4.3)	0.2
No	22/37 (59%)			
**Sex**				
Female	36/46 (78%)	0.1	0.5 (0.2 – 1.3)	0.1
Male	53/82 (65%)			
**First Nation**				
Yes	22/34 (65%)	0.5		
No	58/81 (72%)			
**Alcohol excess**				
Yes	39/66 (59%)	0.02	0.5 (0.2 – 1.1)	0.1
No	42/53 (79%)			
**Diabetes mellitus**				
Yes	39/59 (66%)	0.5		
No	47/65 (72%)			
**Chronic Kidney disease**				
Yes	6/7 (86%)	0.7		
No	74/107 (62%)			
**Malignancy**				
Yes	12/16 (75%)	0.8		
No	73/106 (69%)			
**Lung disease**				
Yes	26/37 (70%)	1		
No	61/87 (70%)			
**No Risk Factors**				
Yes	12/13 (92%)	0.1		-
No	76/112 (68%)			
**Pneumonia**				
Yes	59/88 (67%)	0.3		
No	28/37 (76%)			
**Bacteraemia**				
Yes	58/87 (67%)	0.3		
No	28/37 (76%)			
**Solid organ abscess**				
Yes	58/87 (67%)	0.2		
No	70/104 (67%)			
**Skin & Soft tissue**				
Yes	11/17 (65%)	0.8		
No	72/103 (70%)			
**Genitourinary**				
Yes	13/19 (68%)	1		
No	71/102 (70%)			
**Septic Shock**				
Yes	14/24 (58%)	0.2		
No	65/90 (72%)			

Supplementary Table 7. Bivariate and multivariate associations with *bimA*$_{Bm}$

	bimA$_{Bm}$	Bivariate p-value	aOR (95% CI)	Multivariate p-value
	**N (%)**			
**Age >50**				
Yes	11/91 (12%)	<0.001	0.5 (0.1 – 2.7)	0.4
No	7/37 (19%)			
**Sex**				
Female	11/46 (24%)	0.9		
Male	6/82 (7%)			
**First Nation**				
Yes	11/34 (32%)	<0.001	0.8 (0.2 – 4.6)	0.8
No	7/81 (9%)			
**Alcohol excess**				
Yes	8/66 (12%)	0.001	0.5 (0.2 – 1.6)	0.2
No	9/53 (17%)			
**Diabetes mellitus**				
Yes	8/59 (14%)	0.6		
No	9/65 (14%)			
**Chronic Kidney disease**				
Yes	2/7 (29%)	1		
No	14/107 (13%)			
**Malignancy**				
Yes	2/16 (13%)	0.3		
No	15/106 (14%)			
**Lung disease**				
Yes	4/37 (11%)	0.8		
No	13/87 (15%)			
**No Risk Factors**				
Yes	0/13 (0%)	0.1		-
No	17/112 (15%)			
**Pneumonia**				
Yes	12/88 (14%)	0.05	1.5 (0.4 – 5.8)	0.6
No	4/37 (11%)			
**Bacteraemia**				
Yes	13/87 (15%)	0.4		
No	4/37 (11%)			
**Solid organ abscess**				
Yes	1/15 (7%)	0.3		
No	16/104 (15%)			
**Skin & Soft tissue**				
Yes	1/17 (6%)	0.2		
No	16/103 (15%)			
**Genitourinary**				
Yes	3/19 (16%)	0.8		
No	14/102 (14%)			
**Septic Shock**				
Yes	4/24 (17%)	0.2		
No	13/90 (14%)			
